# Adaptive mechanism in *Quercus brantii* Lindl. leaves under climatic differentiation: morphological and anatomical traits

**DOI:** 10.1038/s41598-023-30762-1

**Published:** 2023-03-03

**Authors:** Forough Soheili, Mehdi Heydari, Stephen Woodward, Hamid Reza Naji

**Affiliations:** 1 Department of Forest Sciences, Ilam University, Ilam, 69315-516 Iran; 2 School of Biological Sciences, University of Aberdeen, Aberdeen, AB24 3UU UK

**Keywords:** Plant ecology, Stomata

## Abstract

Leaf traits, which vary across different climatic conditions, can reveal evolutionary changes within a species made to adapt to the environment. Leaf traits play major roles in a plant functions under varying climatic conditions. To examine adaptive modes and mechanisms applied by plants in different climates, we analyzed leaf morphology and anatomical structures in *Quercus brantii* in the Zagros forests, Western Iran. The plants adapted to the environmental differences with increased dry matter content in a Mediterranean climate, and increasing leaf length, specific leaf area, stomata length (SL), stomata width, stomatal density (SD), stomatal pore index (SPI), trichome length, and width in a sub-humid climate; trichome density was increased in a semi-arid climate. There were strong, positive correlations between SPI with SL and SD. Correlations for other leaf traits were weakly significant. Such morphological and anatomical plasticity probably leads to lower transpiration rates, control of internal temperature and water status, and improved photosynthetic capability under stressing conditions. These findings provide new insights into the adaptive strategies of plants to environmental changes at the morphological and anatomical levels.

## Introduction

The influence of factors affecting the distribution of plant pecies can be inferred the functional traits, and can be used to predict the effects of climate change on ecology and ecosystems^1^. Variations in leaf traits are indications of how plants adapt to the environment based on climatic conditions^2,3^. If climate change effects are exacerbated, these changes can affect forest biodiversity and ecosystem function^4^.

The Zagros mountains, in Iran, with a length of 1600 km from north to south and 240 km from east to west, include large areas of forests and woodlands^5^. Persian oak (*Quercus brantii* Lindl.), a species endemic in Iran, Iraq, Syria, Turkey and West Asia^6^, is the most important tree in the Zagros forest region of western Iran, covering over half of this region^7,8^. Based on the De Marton dryness index^9^, four climates occur in this region, classified as humid, semi-humid, Mediterranean and semi-arid^10^. Dry periods in the first two climatic classifications last for 4–5 months and in the two latter for 4–6 months each year^11^. This region has experienced climate change with warmer temperatures and reduced rainfall which had adverse ramifications for Persian oak trees^12^.

Leaf characteristics at the cellular (e.g., stomatal traits), tissue (e.g., anatomical traits) or organ (morphological traits) levels may be informative with regard to responses to climate since they reflect elements of carbon acquisition, water exchange and gas exchange^13,14^. In investigating relationships between leaves, stems and roots, Wang et al.^15^*.* showed that leaf traits strongly represented overall plant characteristics. Leaf functional traits, therefore, probably contribute to how plants adapt to the environment^16^.

Plants respond rapidly to environmental changes by opening and closing stomata^17^. Stomatal characteristics are modulated during environmental changes^18^, such as light intensity^19^, temperature^20,21^ and plant water status^22^. Trichomes (pubescence) are amongst the important features of plant anatomy involved in tolerance of environmental stresses, including excess transpiration, high temperatures, water deficits and harmful solar radiation^23^. Many aspects of plant functions can be affected by leaf trichomes^24^. Plant trichomes on the leaves have two distinct functions: they act as a structural defense against herbivores^25^ and protect against environmental factors such as solar radiation, including UV and high temperature^26^.

Roy et al.^27^*.* and Wilkens et al.^28^*.* reported a flexible relationship between trichome density and environmental conditions. Plants growing in open, hot, dry habitats tend to have hairier leaves than similar plants or even the same species in more mesic, less exposed habitats^25,29^. Deng et al*.*^30^ also showed that trichome morphology in Fagaceae from temperate forests was controlled by phylogeny and environment. There was a significant correlation between morpho-physiological traits of leaves which suggested that climate played a small role in correlations between the traits considered^31^. According to de la Riva, et al.^32^*.* leaf mass per unit area and nutrient concentrations correlated with characteristics such as phylogeny, habitat and leaf habit in 98 Mediterranean woody species. Moreover, Tian et al*.*^2^ found that climate and leaf nutrients were the main factors regulating morphological and descriptive traits of leaves: they identified a positive correlation between leaf unit area and dry weight and a negative correlation between stomatal length and stomatal density. Functional traits differ between temperate and subtropical forests, with respect to forest type; dry matter content, stomatal density, and cell tense ratio followed the order trees > shrubs > herbs, whereas specific leaf area and sponginess ratio showed the opposite pattern^13^.

The work reported here provides a basis for understanding how climate change affects Persian oak (*Quercus brantii*) trees. Due to their wide distribution, the absolute characteristics of Persian oak differ, depending on region. The hypotheses tested were: (1) the relationships between leaf traits in oak trees are not consistently stable within forest communities and structural performance activity can be affected by climate change; (2) Understanding the impact of climate change on leaf traits can be used to determine what adaptation strategies plants use and can help predict responses in the future; (3) These data makes it possible to determine which leaf characteristics are more variable due to environmental change, as well as provide useful parameters in predicting the impacts of global climate change on trees. Therefore, in order to obtain basic information about this valuable species, the work described here focused on leaf characteristics of *Q. brantii* in relation to habitat changes.

## Results

### Leaf morphological traits under climatic differentiation

**Figure 1:**
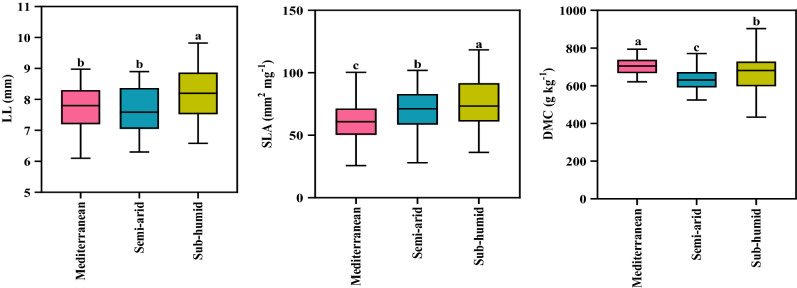
Leaf morphological traits of *Quercus brantii* in three different climates. Leaf length (LL), specific leaf area (SLA), dry matter content (DMC). Vertical bars in each column represent Standard Deviation. Lowercase letters indicate significant differences between three climates (Duncan's multiple range test). The same letters do not significantly differ (*P* < 0.05).

The mean LL and SLA were highest on trees growing in a sub-humid climate (*P* < 0.01.) compared to the other two climate types. Average DMC was significantly higher in the Mediterranean climate than the other two climates (Fig. 1).

### Leaf anatomical traits under climatic differentiation

**Figure 2:**
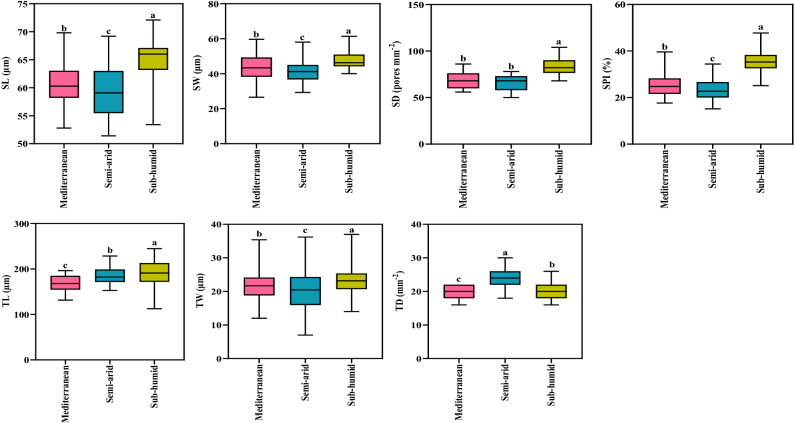
Leaf anatomical traits of *Quercus brantii* in three different climates. Stomata length (SL), stomata width (SW), stomatal density (SD), stomatal pore index (SPI), trichome length (TL), trichome width (TW) and trichome density (TD). Vertical bars in each column represent Standard Deviation. Lowercase letters indicate significant differences between three climates (Duncan's multiple range test). The same letters do not significantly differ (*P* < 0.05).

SL, SW, SD, SPI, TL and TW were significantly higher (*P* < 0.01) in the sub-humid climate compared with the other two climates. TD was highest in the semi-arid climate, but the was no difference between the other two climates (Fig. 2).

### Variety of leaf traits under climate, population and trees

The effect of climate was significant on all leaf traits except for TW. The effect of population was significant on leaf traits except for LL and DMC. The effect of the tree was significant only on TW, SW, DMC and SLA traits (Tables 1 and 2).

**Table 1:** Leaf morphological traits in ANOVA hierarchical model between three different climates.

Source of variation	df	LL (mm)		SLA (mm^2^ mg^−1^)		DMC (g kg^−1^)	
		MS	F	MS	F	MS	F
Climate	2	10.2707**	18.59	7008.0*	4.24	180,660**	18.10
Population (climate)	11	0.5477^ ns^	1.00	1558.1**	3.07	9466^ ns^	1.54
Tree (climate Population)	50	0.5487^ ns^	1.05	492.9**	2.11	6056*	1.50
Leaf (climate population tree)	335	0.5212	–	233.9	–	4042	–
Total	398	–	–	–	–	–	–

LL: Leaf length, SLA: specific leaf area, DMC: dry matter content.

**Table 2:** Leaf anatomical traits in ANOVA hierarchical model between three different climates.

Source of variation	df	SL (μm)		SW (μm)		SD ( pores mm^−2^)		SPI (%)		TL (µm)		TW (µm)		TD (mm^-2^)	
		MS	F	MS	F	MS	F	MS	F	MS	F	MS	F	MS	F
Climate	2	1051.661**	26.68	1395.16**	9.22	10,781.08**	59.06	4928.05**	115.23	18,456.7**	8.33	381.30^ ns^	1.36	724.717**	57.39
Population (Climate)	11	37.682*	2.06	142.97*	2.60	178.07**	2.84	41.74*	2.09	2153.8**	7.21	268.75**	10.70	12.742**	3.68
Tree (Climate Population)	50	18.026^ ns^	1.35	53.70**	1.82	62.88^ ns^	0.93	19.95^ ns^	1.01	305.9^ ns^	0.70	24.67*	1.49	3.625^ ns^	0.55
Leaf (Climate Population Tree)	335	13.378	–	29.51	–	67.35	–	19.82	–	439.1	–	16.56	–	6.589	–
Total	398	–	–	–	–	–	–	–	–	–	–	–	–	–	–

Stomata length (SL), stomata width (SW), stomatal density (SD), stomatal pore index (SPI), trichome length (TL), trichome width (TW) and trichome density (TD).

*P < 0.05; **P < 0.01, ns: non-significant.

### Principal components analysis (PCA) and correlation between leaf functional traits along climatic gradient

**Figure 3:**
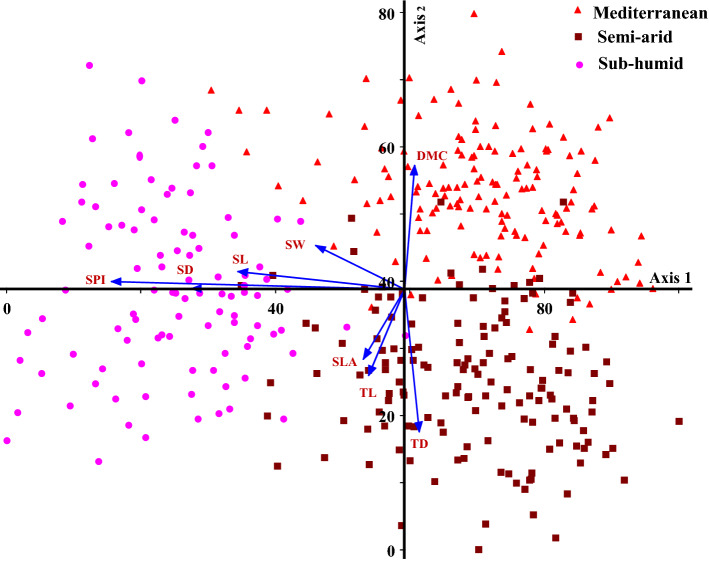
Biplot of principal component analysis (PCA) between *Quercus brantii* leaf morphological and anatomical traits in three different climates. Leaf length, (LL), specific leaf area (SLA), dry matter content (DMC); stomata length (SL), stomata width (SW), stomatal density (SD), stomatal pore index (SPI), trichome length (TL), trichome width (TW) and trichome density (TD).

**Figure 4:**
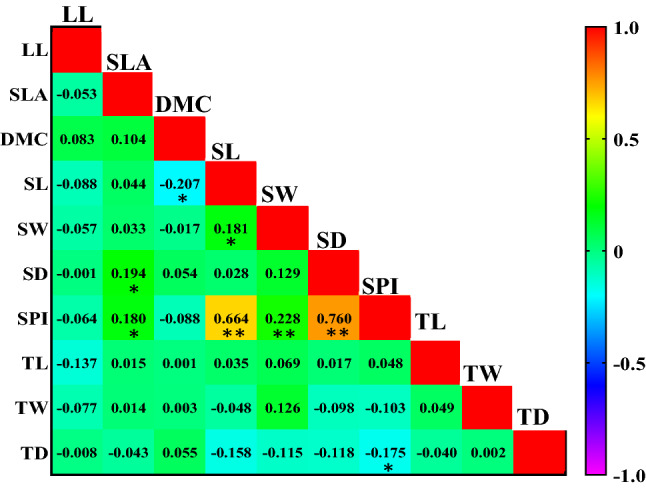
Pearson’s correlation coefficients between leaf morphological and anatomical traits of *Quercus brantii* in Mediterranean climate. Orange and yellow represent strong positive correlations. Leaf length (LL), specific leaf area (SLA), dry matter content (DMC); stomata length (SL), stomata width (SW), stomatal density (SD), stomatal pore index (SPI), trichome length (TL), trichome width (TW) and trichome density (TD). *P < 0.05; **P < 0.01.

**Figure 5:**
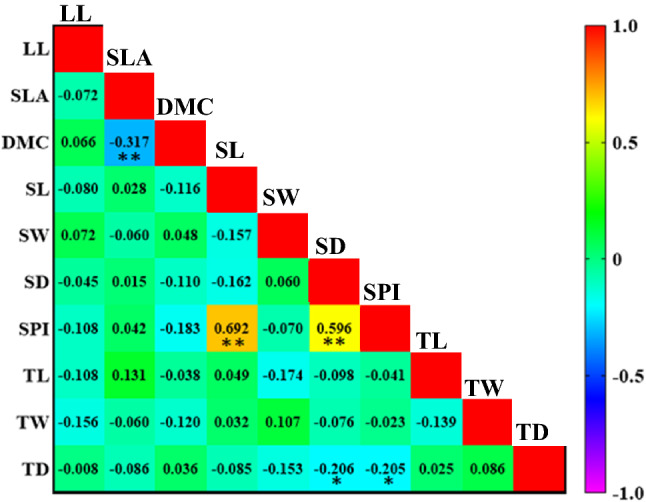
Pearson’s correlation coefficients between leaf morphological and anatomical traits of *Quercus brantii* in sub-humid climate. Orange and yellow represent strong positive correlations, and blue strong negative correlations. Leaf length, (LL), specific leaf area (SLA), dry matter content (DMC); stomata length (SL), stomata width (SW), stomatal density (SD), stomatal pore index (SPI), trichome length (TL), trichome width (TW) and trichome density (TD). *P < 0.05; **P < 0.01.

**Figure 6:**
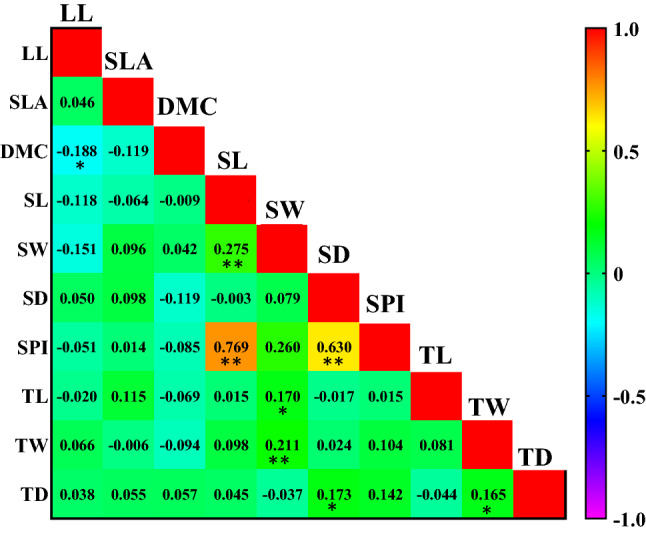
Pearson’s correlation coefficients between leaf morphological and anatomical traits of *Quercus brantii* in semi-arid climate. Orange and yellow represent strong positive correlations. Leaf length, (LL), specific leaf area (SLA), dry matter content (DMC); stomata length (SL), stomata width (SW), stomatal density (SD), stomatal pore index (SPI), trichome length (TL), trichome width (TW) and trichome density (TD). *P < 0.05; **P < 0.01.

The axes first and second PCs explained 29.27 and 15.64% of the variance in morphological and anatomical traits under different climatic conditions. The different climatic regions were separated based on morphological and anatomical traits along axes 1 and 2. DMC was the most important factor in the Mediterranean climate. SL, SW, SD, SPI, TL and SLA were more effective in sub-humid climate. In addition, TD played an important role in distinguishing trees growing the semi-arid climate from other climates (Fig. 3; Table 3). Leaf morphological and anatomical traits showed different correlations in different climates. However, some relationships were observed only in one plant functional group and one climate. In the three different climates, there was a strong and positive relationship between SPI, SL and SD. There was a weak and significant correlation, however, between the other traits in the three climates (Figs. 4, 5 and 6).

**Table 3:** Leaf functional traits in the principal component analyses (PCA) between three different climates.

Trait	Component	
	Axis 1	Axis 2
LL (mm)	− 0.267^ ns^	0.070^ ns^
SLA (mm^2^ mg^−1^)	− 0.353*	− 0.456*
DMC (g kg^−1^)	0.172^ ns^	0.607**
SL (μm)	− 0.706**	0.223^ ns^
SW (μm)	− 0.517**	0.360*
SD ( pores mm^−2^)	− 0.796**	0.057^ ns^
SPI %	− 0.935**	0.155^ ns^
TL (µm)	− 0.327*	− 0.507**
TW (µm)	− 0.260^ ns^	0.223^ ns^
TD ( mm^-2^)	0.206^ ns^	− 0.652**
Eigen values	3.22	1.72
% of Variance	29.27	15.64

LL: Leaf length, DMC: dry matter content, SLA: specific leaf area, SL: stomata length, SW: stomata width, SD: stomatal density, SPI: stomatal pore index, TL: trichome length, TW: trichome width, TD: trichome density. *P < 0.05; **P < 0.01.

## Discussion

Climate change, particularly temperature and precipitation changes, will be of great importance in semi-arid areas^33^. It is possible for climate change to undermine the delicate balance within ecosystems as a result of the impacts on tree species^34^, as tree growth patterns change markedly. Currently, forest ecosystems and tree species are being studied in detail to understand the responses to climate change^33^.

Plants acquire resources from the environment and generally have a high SLA^35^. Water availability is very important and in areas with water scarcity changes occur in plant growth and SLA^36^. In the work reported here, SLA and LL were higher in the sub-humid climate than in the other two climates (Fig. 1; Fig. 3). According to Liu, et al*.*^13^ SLA can reflect the light-capturing potential of leaves; it may be an adaptation to low light intensities that results in plants in temperate forests having higher SLAs, as a lower SLA indicates a higher construction cost. Therefore, adaptation of the leaves to a changing environment may happen through an increase in SLA.

DMC is amongst the key traits in leaf production economics^31^, used to assess plant adaptation and acclimation to environmental conditions^37^. In trees growing in the Mediterranean climate, DMC was higher than plants in the other two climates (Fig. 1; Fig. 3). Leaves with higher DMC are more resistant to moisture diffusion^13^. To adapt to water scarcity, increased DMC is, arguably, the most important strategy. An increase in leaf strength and photosynthetic tissue per square meter is positively related to increasing leaf strength, contributing to higher tolerance to drought conditions^38^. Relatively high DMC in a Mediterranean climate might indicate high tolerance of plants to increased aridity. A similar pattern of results was found in Mediterranean shrub lands^39^ and tropical dry forests under large-scale aridity conditions^40^.

Leaf stomata exchange gases and some particulate matter between plants and the environment, functions that are impacted by the environment^41^. Alterations in stomatal density and size, therefore, influence these exchanges. For example, the larger the stomatal area, the larger channel for gas and moisture exchange^42^. SL, SW, SD were highest in sub-humid climates (Fig. 2; Fig. 3). These findings suggest that, in more humid regions, trees might invest more in structural tissues than in physiologically regulated substances to help cope with water scarcity. Light-limited environments require plants to have large stomata for increased gas exchange and photosynthetic efficiency^17^, in addition, Zhu et al*.*^41^ showed that plants grown in environments with high humidity have larger stomata. The ability of leaves to regulate transpiration is enhanced by a higher stomata density^43^. Kjelgren, et al.^44^*.* stated that plants such as *Dianella revoluta* 'Breeze' ('Breeze' blueberry lily) and *Ptilotus nobilis* (yellow tails) native to arid regions with water deficit exhibited greater reductions in stomatal conductance than those native to humid regions.

Environmental changes affect distribution and size of stomata as key adaptive traits^45^. A higher stomatal density will also more likely cause plants to lose moisture through the leaves, compared with a lower stomatal density^46,47^. SPI reflects both stomatal density and stomatal length; it is an integrated parameter implying a larger stomatal conductance and greater photosynthetic capacity. It is one of the adaptive strategies of leaf stomatal traits to the changing environment. As a result of an increase in SPI, leaves exhibit higher stomatal conductance and photosynthetic capacity^48^, and. SPI was highest in sub-humid climates, compared to the other two climates (Fig. 2; Fig. 3). Liu, et al.^13^*.* showed that, CO_2_ diffusion was reduced at low temperatures, therefore reduced stomatal conductance probably led to a larger SPI in temperate forests.

Trichomes comprehensively reflects the functions of leaves in terms of light and water, and are important plant traits in adaptations to changing environmental conditions. Due to the specific structure of trichomes, the densities, length and distribution all contribute to resistance to natural stresses. TL and TW were highest in sub-humid climates, and TD was higher in semi-arid climates (Fig. 2; Fig. 3). In summary, changing trichome size (length and width) is a significant adaptation to cope with changes of temperature in different environmental. Chen, et al*.*^49^ reported that relationship between leaf reflectance, trichome density and epidermal cell size/density, might indicate changes in cell size. It could be concluded that trichome density predominantly controls leaf reflectance in different environmental conditions. In many oak species, trichomes are amongst the functional strategies to tolerate drought conditions, rainfall regimes and other abiotic stresses^50^. In these species, a high number of trichomes maintains a large leaf boundary layer to reduce water loss. A great density of trichomes is linked to plants adjusted to xeric environments^51,52^ and because pubescent individuals have lower mortality than glabrous individuals after climatic drought events, even within a species^53^. In this sense, trichomes are probably functioning more to increase the boundary zone thickness (/volume) of leaf surfaces and, therefore, reduce water loss. This feature is obviously present in Persian oak. Therefore, leaf morphological and anatomical traits in plant species can be modified to keep the nutrient content at an optimal level in different environmental conditions for a given light and water status.

Correlations between traits may vary as a response to adaptation to changes in the environment. In the sub-humid climate (Fig. 5), there was a significant negative correlation between DMC and SLA indicating water retention ability, because lower SLA may decrease water loss but higher DMC may increase moisture diffusion resistance. Increasing DMC and decreasing SLA is an adaptation in sub-humid climates in order to absorb more moisture and exchange more gas by increasing photosynthetic rate. SPI had a strong positive correlation to SD and SL in three climates (Figs. 4, 5 and 6). However, a weak but significant correlation was observed between other leaf traits. Casson and Hetherington^54^ showed that stomatal density and size directly affected the transpiration and photosynthesis rates of plant leaves. The adjustment of stomatal opening and closing, and optimization of stomatal density and size are key factors associated with plant environmental adaption. Thus, correlation analysis of stomatal characteristics and other variables was of great importance. According to Tian et al*.*^2^ these strong correlations between anatomical traits suggest that plants adapt to changing environmental conditions by adjusting leaf structures. The anatomical characteristics of leaves seem to be strongly related to temperature^55,56^, therefore plants respond to temperature as the strongest factor when adapting to the environment and utilizing resources. Li et al*.*^57^ stated that multifactorial changes in environmental conditions lead to multidimensional adaptation strategies. Whilst forest ecosystems are resilient, climate can shape and shift many species in forest ecosystems, and the findings reported here suggest that trait correlations may vary according to environmental conditions, which may be an aspect to consider regarding global climate change^13^. The ANOVA hierarchical analysis in Tables 1 and 2 showed that the effects of climate and population (environmental variability) were much stronger than the effects of trees (genetic variability and inter-individual phenotypic variability). The results exhibited that environmental variables explained most of the variations in the leaf traits, which is in consistent with the results of An et al.^58^. Furthermore, this result highlights the importance of climate variability in regulating variation in plant functional traits^59–61^.

## Conclusions

Due to the wide distribution of the Persian oak (*Quercus brantii*) trees and the importance of this tree species due to its ecological position in the Zagros forests, it was considered necessary to examine their eco-physiological characteristics. To our knowledge, this is the first study that combined leaf morphological and anatomical traits to explore the adaptation strategies under different climates over a large contiguous forested area. The findings illustrated the fact that plants adapted to environmental differences by increasing dry matter content when growing in a Mediterranean climate, and increasing leaf length, specific leaf area, stomata length, stomata width, stomatal density, stomatal pore index, trichome length and trichome width during growth in the sub-humid climates, whereas trichome density was increased in semi-arid climates. Furthermore, we identified strong correlations of SPI with SL and SD, reflecting the adaptive capacities of leaf morphological and anatomical traits. There was a weak significant correlation observed, however, between other leaf traits. In order to enhance understanding of leaf morphology and anatomical trait variations in the natural environment, we should focus on different plant species in different climates. Furthermore, further research, combining physiology, morphology and gene expression, is required to explore the adaptation strategies in different and changing climates.

## Material and methods

### Study area

**Figure 7:**
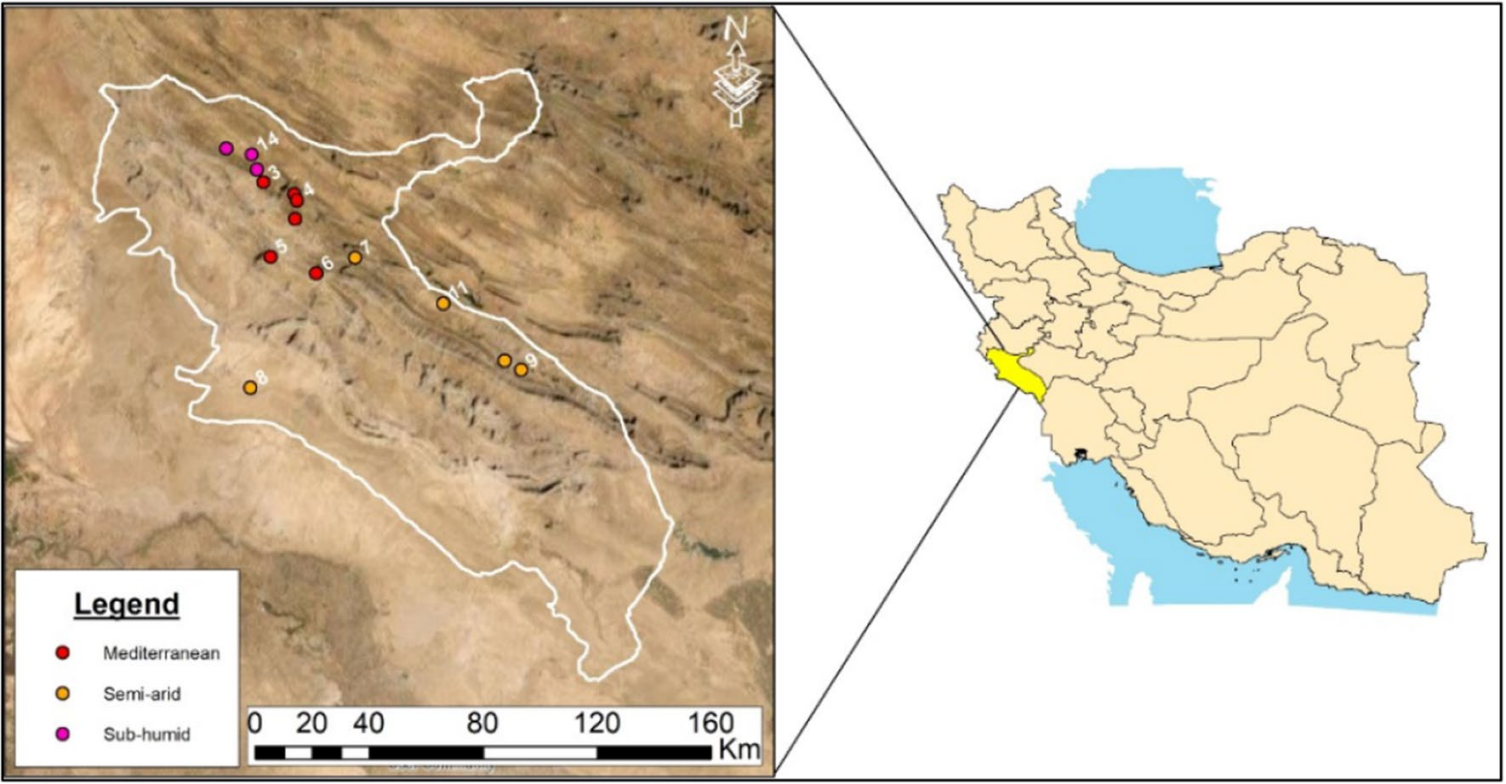
Location of the forests sampled in the Ilam Province in western Iran.

**Figure 8:**
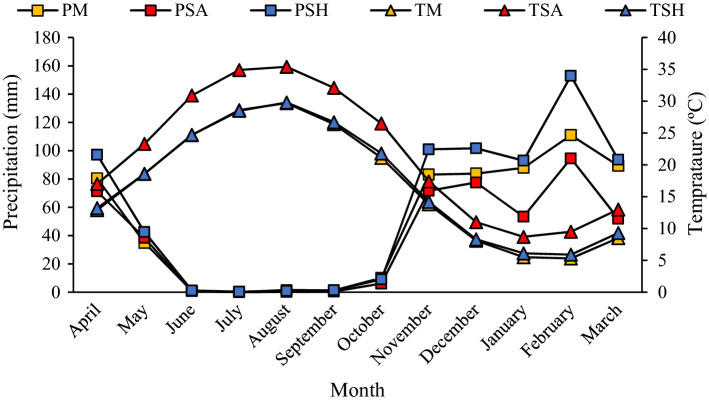
Climate diagrams for parts of the Zagros forests in Ilam province; PM: Precipitation Mediterranean, TM: Temperature Mediterranean, PSA: Precipitation Semi-arid, TSA: Temperature Semi-arid, PSH: Precipitation Sub-humid, TSH: Temperature Sub-humid.

This study was conducted in the Zagros forests of western Iran, the largest oak-dominated forest in the world, covering over 5 million ha, dominated by Persian oak (*Quercus brantii*). Fourteen forest stands across the Ilam province with area of approximately 460 × 10^3^ ha were used in this study. Based on the De Marton dryness index^9^ (Eq. 1), three climatic divisions were delineated in this region (Table 4; Fig. 7). Climate data for various parts of the Zagros forests are given in Fig. 8, showing that the dry period lasts between 4 and 5 months; despite relative good amounts of annual precipitation (average precipitation and temperature were calculated for the period 1987–2021 (Ilam Meteorological Bureau, 2018). The *Q. brantii* resulted from natural regeneration. Sampling was carried out in areas with uniform conditions of habitat, altitude and topography, all in the same biosocial classes. The dominant trees in the forest were *Q. brantii*, *Crataegus sp*., *Acer monspessulanum* and a shrub *Daphne sp*.

1 $${\text{DI}} \, = \, \frac{{\text{MAP}}}{{\text{MAT+10}}}$$

where DI: De Martonne dryness index/MAP: mean annual precipitation; MAT: mean annual temperature.

**Table 4:** Basic characteristics of the *Quercus brantii* forests sampled in this work.

No	Sites	Altitude (m)	Location	Climatic groups	MAP (mm)	MAT (°C)
1	Shalam Ilam	1874	33º 35′ 22″ N 46º 30′ 10″ E	Mediterranean	572	16.9
2	Mian tang Ilam	1648	33º 40′ 03″ N 46º 30′ 00″ E			
3	Dalab Ilam	1409	33º 42′ 24″ N 46º 23′ 01″ E			
4	Sheshdar Ilam	2230	33º 38′ 55″ N 46º 30′ 37″ E			
5	Sad Ilam	1139	33º 28′ 12″ N 46º 24′ 25″ E			
6	Malekshahi	1490	33º 24′ 56″ N 46º 34′ 38″ E			
7	Pakal Dareh shahr	1261	33º 27′ 46″ N 46º 43′ 38″ E	Semi-arid	466.5	21.5
8	Dareh shahr	933	33º 3′ 30″ N 46º 19′ 30″ E			
9	Sarab Dareh shahr	826	33º 05′ 52″ N 47º 20′ 38″ E			
10	Kabir koh Dareh shahr	1075	33º 07′ 41″ N 47º 16′ 59″ E			
11	Badreh Dareh shahr	1100	33º 18′ 50″ N 47º 03′ 26″ E			
12	Khoran Eyvan	1337	33º 48′ 55″ N 46º 14′ 41″ E	Sub-humid	685.7	17.2
13	Sarab Eyvan	1437	33º 44′ 51″ N 46º 21′ 37″ E			
14	Chalanchi Eyvan	1492	33º 47′ 40″ N 46º 20′ 26″ E			

MAP (mm): Mean annual precipitation, MAT (°C): Mean annual temperature.

### Sampling method

Leaf samples from *Q. brantii* were collected during July and August 2021. Initially, one plot (100 × 100 m) was established in each area. In each forest stand, five seed-derived trees (total 70 trees), with diameter at breast height (DBH) ranging from 30 to 40 cm were selected. Experimental research on plants including the collection of plant material complies with relevant institutional and national guidelines and legislation under Permission No.: 1400/S/11,544 from Bureau of Ilam Forest and Watershed. The trees had no obvious signs of damage, disease and crown dieback. Geographic/location information (latitude, longitude, altitude), plant species composition and ecosystem structure were recorded for each plot. Leaves were collected using a telescopic branch pruner from the outer-middle part of the tree crown. Thirty fully-matured, sun-light exposed leaves were collected from five individual *Q. brantii* in each forest stand. All leaf samples from each forest stand were then mixed with each other, representing one replicate^62^. Immediately after collecting, leaves were put into plastic bags and placed in a cold box at approx. 4 °C. To better conserve the leaves, samples were fixed in FAA (50%; alcohol: formalin: glacial acetic acid: glycerin = 90:5:5:5 v/v)^13^.

### Measurement of leaf traits

#### Morphological traits

After sampling, leaf length (LL, mm), was measured using digital calipers (INSIZE) an accuracy of 0.02 mm. Leaf area (mm^2^) was measured using a leaf area meter (CI-202, CID Bioscience, USA,). Fresh weight (LFW, mg per leaf) was recorded on an A&C -320.3 balance (3 Accu LAB, Germany) t an accuracy of 0.0001 g; leaves were subsequently dried to constant weight in an oven at 60 °C^63^ to measure leaf dry weight (LDW, mg per leaf). Dry matter content (DMC, g kg^−1^), and special leaf area (SLA, mm^2^ mg^−1^) were calculated using the Equations^13^:

2 $$ {\text{DMC}} = {\text{LDW/LFW }} \times {1000} $$

3 $${\text{SLA}}= {\text{ LA/LDW}}$$

### Anatomical traits

In order to measure leaf anatomical traits, due to high density of trichomes and to better visualize of the epidermal layer, the density of trichomes was reduced using a twin-blade razor and adhesive tape. Chlorophylls were extracted by immersion in a mixture (1.5:100 v/v) pf acetic acid (99%) and hydrogen peroxide (30%), respectively at 100 °C for one hour in a Bain-Marie water bath^64^. After twice washing in distilled water, samples were stained in aqueous safranin O before dehydrating in an ethanol series (60%, 85%, 95% and absolute for 15 min), before mounting on glass microscope slides in Canada balsam. Finally, 30 stomata and trichomes were randomly selected to measure stomata length (SL, μm), stomata width (SW, μm), trichome length (TL, μm) and trichome width (TW, μm) using optical image analysis (True chrome metrics, Fuzhou, China) attached to a computer. The arms of compound trichomes (fasciculate and stellate) were measured from the point of attachment (excluding the pedestal of fasciculate trichomes) to the distal end of the arms^65^. The number of stomata (SD) and the number of trichomes (TD) in a field of 0.5 mm^2^ was calculated using Eq. 4^66^, while stomatal pore index (SPI) was calculated with Eq. 5^48^:

4 $${\text{SD \, and \, TD}} = \frac{\text{N}}{0.5}$$

where 0.5 is the area of leaf surface examines (mm^2^).

5 $$ {\text{SPI}} = {\text{SD}} \times {\text{SL}}^{{2}} \times 10^{- 4} $$

Table 5 shows the descriptions of leaf morphological and anatomical traits of *Quercus brantii* and their strategies in the leaf.

**Table 5:** Abbreviations, units, and description of morphological and anatomical leaf traits.

Traits	Abbr	Unit	Group	Strategy
Leaf length	LL	Mm	Morphology	Resource capture and defense
Special Leaf Area	SLA	mm^2^ mg^−1^	Morphology	Resource capture and defense
Dry Matter Content	DMC	g kg^−1^	Morphology	Resource capture and defense
Stomata Length	SL	Μm	Anatomy	Gas exchange, *e*.*g*. CO_2_ and H_2_O
Stomata Width	SW	Μm	Anatomy	Gas exchange, *e*.*g*. CO_2_ and H_2_O
Stomata density	SD	pores mm^−2^	Anatomy	Gas exchange, *e*.*g*. CO_2_ and H_2_O
Stomata pore index	SPI	%	Anatomy	Gas exchange, *e*.*g*. CO_2_ and H_2_O
Trichome length	TL	Μm	Anatomy	Resource capture and defense
Trichome width	TW	Μm	Anatomy	Resource capture and defense
Trichome Density	TD	mm^-2^	Anatomy	Resource capture and defense

Abbr: Abbreviation.

### Data analysis

Raw data were tested for normality using Kolmogorov–Smirnov test and homogeneity of variances was examined using Levene's test. Means and standard errors of the morphological and anatomical traits were calculated for the three climatic regions, and tested for differences using ANOVA and Duncan’s multiple comparison tests. Heat maps of the correlations between leaf morphological and anatomical traits in the different climates were developed based on Pearson’s correlation coefficients. The statistical software package SPSS 21 was employed for all statistical analyses. Principal component analyses (PCA), based on the correlation matrix, using PC-Ord version 5.0 were used to examine multivariate correlations (i.e. relationships between leaf morphological and anatomical traits across different climatic conditions). For better interpretation of the data, only principal components 1 and 2 (first and second axes) were used. Analysis of variance of leaf traits was calculated with the ANOVA hierarchical model using Minitab software (version 14). The climate, population (nested in climate), and tree (nested in climate and population) were included into the model as climate was assumed a fixed factor and population, tree and leaf were random factors.

## Data Availability

The datasets used and analyzed during the current study available from the corresponding author on reasonable request.
